# Characterization of Copper(II) Interactions with Sinefungin, a Nucleoside Antibiotic: Combined Potentiometric, Spectroscopic and DFT Studies

**DOI:** 10.1155/2007/53521

**Published:** 2007-12-13

**Authors:** Maria Jaworska, Piotr Lodowski, Ariel Mucha, Wojciech Szczepanik, Gianni Valensin, Massimo Cappannelli, Małgorzata Jeżowska-Bojczuk

**Affiliations:** ^1^Department of Theoretical Chemistry, Faculty of Mathematics, Physics and Chemistry, University of Silesia, 14 Bankowa St., 40-007 Katowice, Poland; ^2^Department of Bioinorganic and Biomedicinal Chemistry, Faculty of Chemistry, University of Wrocław, F. Joliot-Curie 14, 50-383 Wrocław, Poland; ^3^Department of Chemistry, Faculty of Mathematical, Physical, and Natural Sciences, University of Siena, Via Aldo Moro, I-53100 Siena, Italy

## Abstract

Interactions between sinefungin and copper(II) ions were investigated. Stoichiometry and stability constants of the metal-free system and two mononuclear complexes present in solution were determined on the basis of potentiometric data analysis. The results were compared to the Cu(II)-ornithine system due to structural similarities between both molecules. Combined spectroscopic and theoretical studies allowed for determination of coordination pattern for the Cu(II)-sinefungin complexes. At acidic pH, copper is bound in “glycine-like” coordination mode, identical with that of ornithine. This involves α-amino group and the carboxyl oxygen. At higher pH, a “bis-complex” is formed by two sinefungin molecules. The second ligand binds in equatorial position displacing two water molecules, what results in the stable {2N,2O} coordination. Both axial positions are supposed to be occupied by N1 nitrogen donors of adenine moiety, what is confirmed by DFT calculations. They interact indirectly with copper(II) through water molecules as the result of dominant *syn* conformation of purine.

## 1. INTRODUCTION

**Figure 1:**
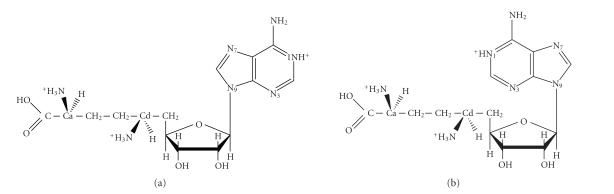
The fully protonated molecule of sinefungin in its two conformers, (a) *anti* and (b) *syn*.

Nucleobases and nucleosides, the structural particles of nucleotides, play relevant roles in several metabolic processes. It is, therefore, not surprising that their analogs are investigated as potential therapeutic agents [1–3]. Sinefungin (SFG, Figure 1), an antifungal and antiparasitic nucleoside antibiotic, is a natural product of *Streptomyces griseolus* and *S. incarnatus* [4, 5]. SFG is active against a number of protozoan [6], inhibits tumor cell invasion in vitro [7], as well as virus multiplication [8], and cell transformation [9]. Structurally, SFG represents a wide range of compounds that combine aminoacids with nucleobases [10–14].

SFG comprises ornithine and adenosine nucleoside residues. Ornithine is well known to act as a precursor in one of the two possible routes to the biosynthesis of putrescine [15], the source of the diaminobutane residue for spermidine and spermine [16, 17]. Both these biogenic amines take part in many biological processes [18–20] in almost all living and even cancer cells [21]. The search for ornithine analogues that inhibit polyamine biosynthesis [22, 23] or act as new anticancer drugs [24] as well as the continuous research on biological activity of various derivatives of nucleosides confirm the importance of the moieties combination in SFG.

Transition metal ions play fundamental roles in biological processes. It has been widely accepted that copper is an essential trace element which forms an integral component of many important enzymes and is required for growth and development in a wide range of species, from bacteria to man [25–27]. However, coordination of metal ions to bioactive ligands may also affect their toxicity. In fact, some metal complexes (e.g., Fe(II) and Cu(II)) of organic compounds used as drugs [28–30] were found to be less toxic than the “native” forms, while maintaining the medical activity. On the other hand, metal complexes (particularly Cu(II)) of pharmacological agents, when compared with the free ligands, are widely reported to generate enhanced toxic effects [31, 32].

Significant amounts of free copper ions are unlikely to occur in vivo [33–35], what was also ratified by the “fingerprint” of LDL oxidation products in extracts from atherosclerotic lesions, which is not consistent with the model of induction by free metals in vitro [36]. However, advanced lesions contain products consistent with free metal ion-catalyzed oxidation [36, 37]. Furthermore, some reports indicate that the concentration of a mobile fraction of copper ions in blood serum may increase as a result of a pathological state (e.g., harmful oxidative stress [38]), cancer, and inflammation [26]. Extracellular copper remains bound to a variety of low-molecular-weight ligands [39, 40], and its control is not as tight as that of the intracellular one, regulated by a series of chaperone proteins [35]. Cupric ions were also found in nuclei in association with chromosomal DNA [41]. It is then possible that some extracellular copper is transferred to SFG, what prompted us to perform the present study, which continues our research concerning the effect of Cu(II) on in vitro toxicity of drugs [32, 33].

Our previous papers reported the results of NMR investigations [42] as well as the oxidative activity of copper complexes with SFG in the presence of biologically important substances [43], and now we provide the detailed information about the coordination mode of copper-sinefungin complexes and their structures.

## 2. RESULTS AND DISCUSSION

Adenine and the other purine nucleobases, as well as their nucleosides and nucleotides, are known to undergo self-aggregation through stacking of aromatic moieties [44–46]. However, at the concentrations used in this work (i.e., 0.7 MM), no self-association of the adenine ring of SFG is expected. This also holds for measurements concerning the metal ion complexes. As a consequence, the reported results all refer to the monomeric species.

### 2.1. Acid-base properties of sinefungin

SFG contains several potential donatives sites for metal ions and/or protons. Precise potentiometric titrations led to characterizing the acid-base properties of SFG, which behaves as a tetraprotic species. Table 1 shows the calculated values of the protonation constants that fit the range of pH 3–10 and considerably differ from those of ornithine (also reported in Table 1).

The following deprotonation equilibria need then to be considered (L stands for SFG):

(1) $$\begin{array}{cc} {\text{H}}_{4}{\text{L}}^{3+} & ⇌{\text{H}}_{3}{\text{L}}^{2+}+{\text{H}}^{+}, \end{array}$$

(2) $$\begin{array}{cc} {\text{H}}_{3}{\text{L}}^{2+} & ⇌{\text{H}}_{2}{\text{L}}^{+}+{\text{H}}^{+}, \end{array}$$

(3) $$\begin{array}{cc} {\text{H}}_{2}{\text{L}}^{+} & ⇌\text{H}\text{L}+{\text{H}}^{+}, \end{array}$$

(4) $$\begin{array}{cc} \text{LH} & ⇌{\text{L}}^{-}+{\text{H}}^{+}. \end{array}$$

On the basis of mathematical analysis of the potentiometric titrations, four ${\text{pK}}_{\text{a}}$ values were calculated. The exact description of the stepwise protonation constants of the acidity of the individual groups is not fully possible. All obtained results give, however, only the acidity sequence of particular groups in the molecule but not the exact acidity constants due to the possibility of the parallel overlapping of the deprotonation processes.

**Figure 2:**
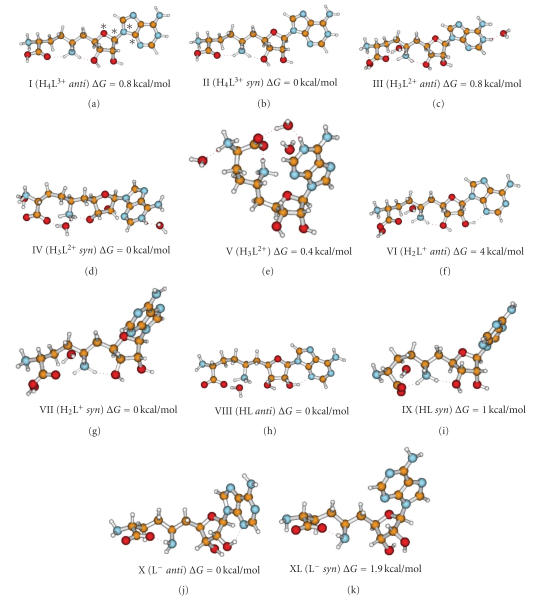
Conformations of free SFG molecule in various protonation states. The rotation angle of adenine is marked with asterisks.

A constant corresponding with the equilibrium (1), the most likely, relates to the release of proton from the carboxylic group, which is the most acidic function of SFG. The pK value of this process $\left({\text{p}\text{K}}_{{\text{H}}_{4}{\text{L}}^{3+}}^{\text{H}}=3.15\right)$ is more than one log unit higher than that found in L-ornithine [47, 48], most probably due to electrostatic interaction with the solvent, with eventual formation of a hydrogen bond between the carboxyl group and water molecules. It should be mentioned that purine nitrogen can be involved in the formation of hydrogen bond with carboxyl hydroxylic group ($\text{OH}\cdots \;\cdots {\text{N}}_{7}$). However, this explanation is valid in the case of *anti* conformation for H_4_L^3+^ species only. As a matter of fact, computational studies concerning conformation of free-SFG indicate that, in the case of H_4_L^3+^ *syn,* conformation predominates (Table 2, Figure 2, structure II), thus increasing the probability of occurrence of an intramolecular hydrogen bond with the protonated N_1_ atom. Such situation would have the reflection in the lower-${\text{pK}}_{\text{a}}$ value of either OH_COOH_ or N_1_(H^+^), however, is not observed in any of these cases. Therefore, the presence of the intermolecular hydrogen bond between solvent water molecule and −OH group is proposed.

The second ${\text{pK}}_{\text{a}}$ value (3.83, Table 1, equilibrium (2)) belongs to N_1_ of the adenine moiety and is very close to the values obtained for adenine monophosphates ($\text{p}{\text{K}}_{AMP\prime s}^{H}=3.84$ [49, 50]). However, it is interestingly higher than that in the case of adenosine (3.61 [45]), in spite of the presence of a bipositive charge within the ornithine residue. Again, a macrochelate-like electrostatic interaction between the carboxyl (deprotonated) and protonated N_1_ nitrogen–N_1_(H^+^) suggests that the *syn* conformer of SFG is preferred. Indeed, theoretical studies (see Figure 2, structure IV, and Table 2) indicate that, for this case (as well as for H_4_L^3+^), the *syn* conformation prevails and the stabilizing intermolecular hydrogen bonds between ${{\text{O}}_{\text{COO}}}^{-}\cdots \;\cdots {\text{H}}_{{\text{H}}_{2}\text{O}}$ and ${\text{O}}_{{\text{H}}_{2}\text{O}}\cdots \;\cdots \text{H}{\left({\text{N}}_{1}\right)}^{+}$ are present. As a result, an *outer-sphere* macrochelate is formed (conformation V in Figure 2), with consequent shift of N_1_ nitrogen pK value. Analogous phenomena involving N_7_ of purine were observed during macrochelate formation in the molecules of $5^{\prime }$-ATP [51] and $5^{\prime }$-dGTP [52].

Next deprotonations can be attributed to the both remaining amino groups (${\text{pK}}_{\text{a}}$ = 8.35 and 9.56, equilibria (3) and (4), resp.). The first, assigned to –${{\text{N}}_{\alpha }{\text{H}}_{3}}^{+}$, is 0.4 log unit lower than the corresponding value for ornithine [47, 48], again, as a consequence of the mentioned macrochelate formation. The second, assigned to –${{\text{N}}_{\delta }{\text{H}}_{3}}^{+}$, is significantly different from the values reported for free ornithine. Such increase in acidity is most likely caused by the presence of the adenine moiety. In fact, nucleobases have similar effects in nucleotides, namely, ${\text{pK}}_{\text{a}}$ value of the second terminal hydroxyl group in AMP ($\text{p}{\text{K}}_{\text{H}\left(\text{AMP}\right)}^{\text{H}}=6.21$ [49, 50]) is lower than the corresponding unaffected value in ATP (${\text{p}\text{K}}_{{\text{H}}_{2}{\left(\text{ATP}\right)}^{2-}}^{\text{H}}=6.47$ [51]). Thus electron withdrawing by the adenine residue may yield the observed decrease in ${\text{pK}}_{\text{a}}$ of −${{\text{N}}_{\delta }{\text{H}}_{3}}^{+}$.

The conformational analysis performed on all the four SFG forms demonstrates that the solvent affects the relative stability of each conformer, thus indicating differences in polarity. The solute-solvent interaction depends on (i) the solute and the distributions of (ii) electronic and (iii) nuclear charge. The electrostatic free energy of solvation (see Table 2) is simultaneously dependent on total charge and polarity of the whole molecule, being the energy of hydratation, larger for ionic than for neutral molecules. The polarized solute-solvent interaction energies change from −326 kcal/mol (for H4L^3+^) to −47 kcal/mol (for neutral HL^±^). The increase of the total polarity of the molecule yields a corresponding decrease in the ligand-solvent interaction energy, with consequent effects on the conformation of the ligand. Such effects are indeed observed for two forms of SFG (i.e., HL^±^ and L^−^), and were ratified by calculating the electrostatic potentials (see supplementary data, Figure 9) of HL^±^ and L^−^ in *syn* and *anti* conformations. The electrostatic potential of HL^±^ is of course much more positive. In the *syn* conformation, there are areas of negative electrostatic potential near the −NH_2_ group, the N_3_ atom of adenine, and the −OH group of ribose moiety, but in the *anti* conformation, the same areas are reduced and the potential is lower as a result of the internal H-bond between N_3_ and OH. In the *syn* conformation of L^−^, there are negative potentials in the vicinity of the −NH_2_ group and the N_3_ atom, which are quite close to each other and destabilize this conformation. The calculations show that the *syn* conformation displays larger electrostatic free energy of solvation than the *anti one* (from *ca.* 3 to 8 kcal/mol). It is obvious that the equilibrium conformations of nucleotides are affected by solvent effects and particularly by intramolecular H-bonds, as reported previously [53]. In the calculations related to SFG, the *syn* conformation is favored in all cases, but for HL and L^−^, the solvation energies indicate differences in polarity.

The rotational barrier of adenine moiety was calculated only for H_4_L^3+^ at 1.4 kcal/mol for *syn*-antirotation and 2.8 kcal/mol for anti-*syn* rotation. This value is very close to the energy barrier for *syn*-*anti* conformation (1.43–1.19 kcal/mol) estimated for cytidine derivatives in aqueous solution at 25°C [54, 55]. The calculated anti-*syn* barrier for fully protonated SFG is over 1 kcal/mol larger than that measured for cytidine derivatives.

The conformations obtained for the diversely protonated forms of SFG are shown in Figure 2. The relative Gibbs free-energy values obtained after addition of the thermal corrections, and the energies of solvation are collected in Table 2, together with the dihedral angle defining the rotation of adenine (atoms defining the rotation angle are marked with asterisks in Figure 2). Boltzmann factors (in percentage) calculated from free energies are also shown in Table 2.

### 2.2. Copper(II) coordination pattern to sinefungin

The process of copper(II) binding by sinefungin as well as similar agents representing the combination of nucleosides and aminoacids, may have biological consequences. As we have already provided, the Cu(II)-sinefungin complexes may be regarded as genotoxic compounds since they cleave DNA with the hydroxyl radicals-based mechanism [43]. DNA degradation proceeds even in the presence of physiologically widespread antioxidants (glutathione, ascorbate), what additionally confirms high toxicity of the studied complexes. Moreover, these studies presented sinefungin as a ligand able to compete for Cu(II) coordination with cellular substances. This result prompted us to undertake current studies aimed to resolve the complexation equilibria in Cu(II)-sinefungin system.

The potentiometric titrations of the Cu(II)-SFG complex allowed to calculate the stability constants of the two mononuclear species, CuHL^2+^ and ${\text{Cu}{\text{H}}_{2}{\text{L}}_{2}}^{2+}$, the formation of which corresponds to equilibria (5) and (6):

(5) $$\begin{array}{cc} {\text{Cu}}^{2+}+\text{HL} & ⇌{\text{CuHL}}^{2+}, \end{array}$$

(6) $$\begin{array}{cc} {\text{Cu}}^{2+}+2\text{HL} & ⇌{{\text{CuH}}_{2}{\text{L}}_{2}}^{2+}. \end{array}$$

The obtained values are reported in Table 1 and compared to the literature data related to ornithine.

**Figure 3:**
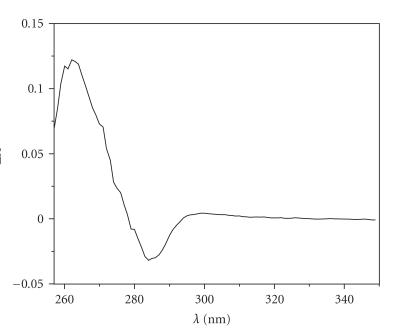
The differential UV spectrum for the Cu(II)-SFG system obtained by subtraction of the spectrum at pH 3.45 from the spectrum at pH 4.57.

**Figure 4:**
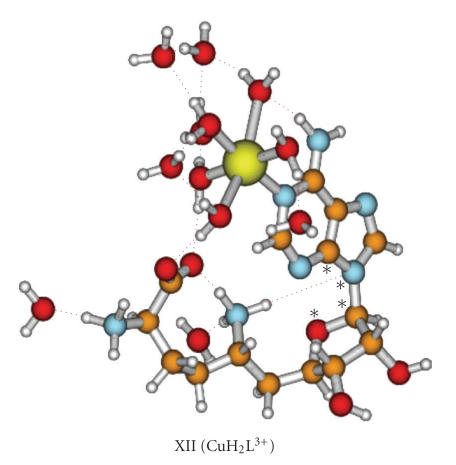
The lowest energy structure of CuH_2_L^3+^ complex. The angle O–C–N–C defining the position of adenine group is marked with asterisks.

Besides the two mentioned species, the eventual formation of CuH_4_L^5+^, CuH_3_L^4+^, and CuH_2_L^3+^ may be discussed. In the CuH_4_L^5+^ complex, Cu(II) should bind at N_7_ of the adenine ring, what is quite improbable because of the electrostatic repulsion of the positive charge at the N_1_(H)^+^ nitrogen. In the CuH_3_L^4+^ form, Cu(II) may bind to the carboxylate, but the log⁡K of this complex is too low to be detected by potentiometry. The case of CuH_2_L^3+^ is somehow different since Cu(II) may directly and simultaneously bind at N_1_ (or N_7_) and the carboxylate. Potentiometric data do not allow to extract the stability constant for this complex, but the differential UV spectra (Figure 3) display a band at *ca.* 260 nm, which is consistent with the interaction of the adenine ring with Cu(II) [56]. Experimental data are not sufficient to demonstrate any stabilizing interaction between the metal-coordinated exocyclic nitrogen donor atom and the COO^−^ group. However, DFT calculations supported the occurrence of a complex, where Cu(II) interacts with the carboxylate through one of the five metal-bounded water molecules (*outer-sphere* macrochelate). The optimized lowest energy structure of CuH_2_L^3+^ is shown in Figure 4, while bond distances and the O–C–N–C angle defining the position of the adenine group are collected in Table 3 (structure XII). The stability of this complex is higher in calculations implying solvent molecules. Hence we can conclude that the complex in this particular structure can occur in solution. The remaining proposals for the CuH_2_L^3+^ complexes are much less stable, and thus they are not included in Table 3 and Figure 4.

**(i) CuHL^2+^ complexes**

**Figure 5:**
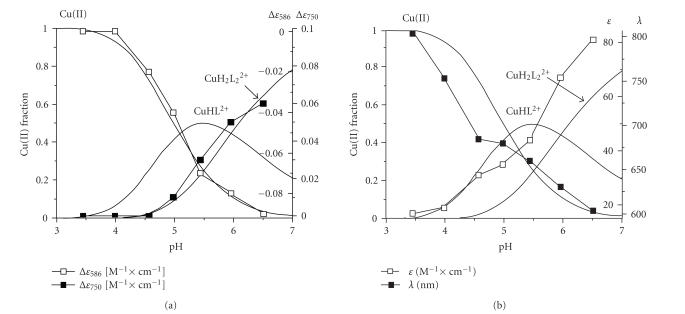
The pH-dependent courses of complexes concentrations and the (a) CD and (b) UV-Vis spectra parameters overlaid.

**Figure 6:**
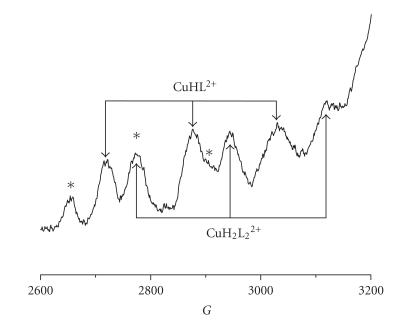
The EPR spectra for the Cu(II)-SFG system at pH 5.6. Signals for complex species are indicated by arrows and signals for Cu(II) aqua ion are depicted with asterisks.

The CuHL^2+^ species, which dominates between pH 5.0 and pH 6.0 (Figure 5), involves Cu(II) binding at the α-amino group and the carboxylate, with consequent formation of a stable 5-membered chelate ring similar to the Cu(II)-ornithine system [47, 48]. Participation of one nitrogen donor in the metal coordination sphere is ratified by the localization of d–d bands on CD and UV-Vis spectra (see Figure 5) as well as the values of EPR parameters $\left({\text{A}}_{\text{II}}=160\;\text{G,}\;\;\;{\text{g}}_{\text{II}}=2.31\right)$ (Figure 6). On the parallel part of the EPR spectrum obtained at pH 5.6 apart from the signals corresponding to the CuHL^2+^ species, also signals derived from the presence of bis-complex with stoichiometry ${\text{Cu}{\text{H}}_{2}{\text{L}}_{2}}^{2+}$ are observed. However, appropriate parameters of the hyperfine splitting can be clearly attributed to the corresponding complex species. Taking into account that, at these conditions, not all copper(II) ions are complexed by SFG molecules, signals for Cu(II) aqua ion are also identified (asterisk in Figure 6). EPR results in this case well reflect the distribution of the copper(II) forms in solution, where free metal ions coexist with both coordination species at pH 5.6 (Figure 5). These results are consistent with the 1N coordination type, although indirect *outer-sphere* participation of the N_7_ donor in the coordination process cannot be excluded [42].

**Figure 7:**
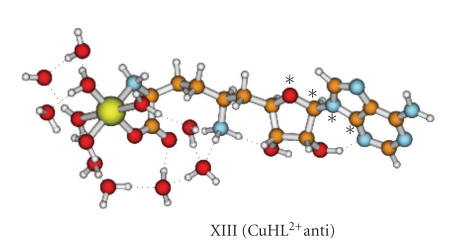
The lowest energy structure of CuHL^2+^ complex. The rotation angle of adenine is marked with asterisks.

The optimized geometry of the lowest-energy structure of CuHL^2+^ is shown in Figure 7, and the corresponding selected geometry parameters are collected in Table 3 (structure XIII). The more extended surface of the anticonformer accounts for larger solute-solvent interactions and such effect energetically favors the *anti* conformation in solution. In this case, the solvation energy (not shown in Table 3) is larger for the anticonformer. As presented in Table 3, the axial water molecules are rather loosely bound, what results in Cu−OH_2_ distance in the range 2.3–2.5 Å. The equatorial water ligands are considerably more strongly bound (the Cu−OH_2_ distance is *ca.* 2.0 Å).

**(ii) ${\text{Cu}{\text{H}}_{2}{\text{L}}_{2}}^{2+}$ complexes**

In the ${\text{Cu}{\text{H}}_{2}{\text{L}}_{2}}^{2+}$ complex, two SFG molecules are bound to the metal ion in the same way as in the previous species (i.e., by α-amino and carboxyl groups) with formation of two stable 5-membered chelate rings. Again, this coordination mode is ratified by spectroscopic data. EPR parameters $\left({\text{A}}_{\text{II}}=180\;\text{G,}\;\;\;{\text{g}}_{\text{II}}=2.26\right)$ reveal the presence of two nitrogen donors (Figure 6) and the changes in CD and UV-Vis spectra (see Figures 5(a), 5(b), resp.) indicate the $\left\{2\times {\text{NH}}_{2},2\times {{\text{O}}_{\text{COO}}}^{-}\right\}$ coordination mode. Participation of N_1_/N_7_ atom in the coordination may occur through *outer-sphere* interactions with axially coordinated water molecules. However, taking into account that *syn* conformation of adenine moiety dominates (see DFT results), we can suppose that N_1_ nitrogen interacts indirectly with Cu(II) ion.

**Figure 8:**
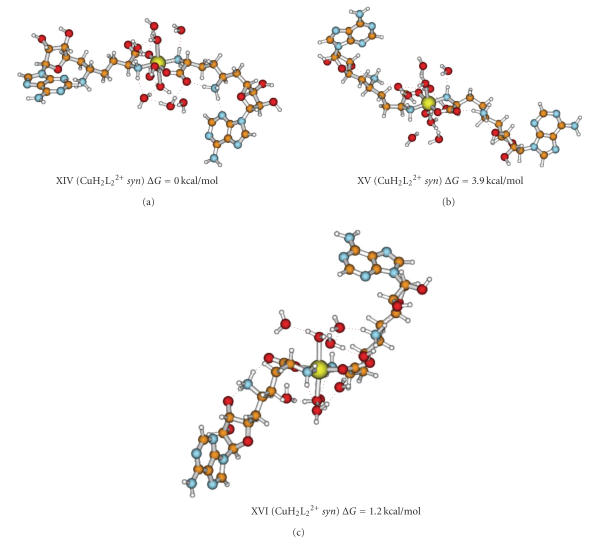
The lowest energy structures of ${\text{Cu}{\text{H}}_{2}{\text{L}}_{2}}^{2+}$ in *syn* conformation. The angle Cu–N–C–C defining the conformation of SFG at the copper center is marked with asterisks.

**Figure 9:**
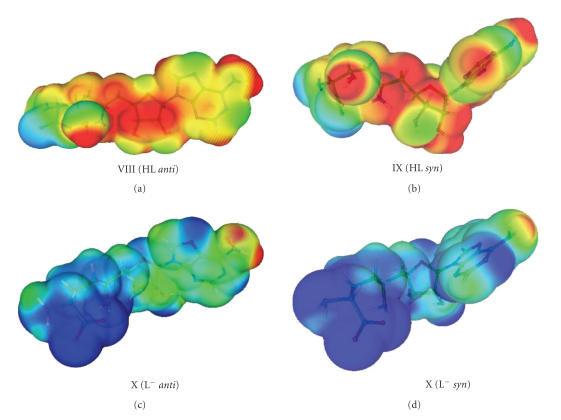
Electrostatic potentials of HL and L^−^ in the *syn* and *anti* conformations. The potential values are in the range from −0.012 [a.u.] (blue) to 0.05 [a.u.] (red).

The proposed coordination mode, that is, two carboxylate oxygen atoms, two *α*-amino group, and two axial water molecules, leading to six-coordinated Cu(II) ion, was taken into account for calculations. The isomers with O–Cu–O and N–Cu–N *trans* and *cis* arrangement were also considered in the optimization with the former ones appearing to be more stable. The geometry optimization was performed for the *syn* and *anti* conformations of the adenine group, and the stability was found somehow higher with the *syn* rather than the *anti* conformation. The three most-stable *syn* conformers are reported in Figure 8. The corresponding free energies, Boltzmann factors and selected structural parameters, are collected in Table 3. The Cu–N–C–C angle defines the conformation of the COO–CH_2_−N_*α*_H_2_ fragment coordinated to copper(II) ion. The angles of the two HL ligands are of the same or opposite sign, what determines the geometry around metal ion: *C_2_* local symmetry in the case of identical sign and *C_i_* in the other case. The formation of intramolecular hydrogen bonds can be noted in all conformers: between −${\text{N}{\text{H}}_{3}}^{+}$ and carboxylate, −${\text{N}{\text{H}}_{3}}^{+}$ and ribose moiety (structures XIV-XVI, Figure 8), as well as −${\text{N}{\text{H}}_{3}}^{+}$ and N_3_ (structure XIV, Figure 8). The formation of a hydrogen bond between the −${{\text{N}}_{\delta }{\text{H}}_{3}}^{+}$ group and the N_3_ nitrogen atom of adenine moiety is only found for structure XIV. This may be a result of different ornithine carbon chain conformation as compared with the remaining structures. In this conformation, adenine and −${\text{N}{\text{H}}_{3}}^{+}$ are close to each other.

The *syn*-*anti* equilibrium of adenine in both free and complexed SFG depends significantly on solvation effects and formation of intra and intermolecular H-bonds in such a manner that small perturbations may result in the meaningful changes. Solvent effects have been found to stabilize the *syn* conformation of adenine in almost all studied systems. However, comparison of the structures I–XVI shows that other factors, like Cu(II) coordination mode, also influence the relative *syn-anti* stability. Isomerism related to the presence of N_1_/N_7_ in the coordination sphere, in the case of both CuHL^2+^ and ${\text{Cu}{\text{H}}_{2}{\text{L}}_{2}}^{2+}$ species, results from the formation of *open* and *close* complexes divided into *outer-* and *inner-sphere*, and does not allow ascribing the spectroscopic parameters to particular complexes.

Molecular features of the complexes that include fully deprotonated SFG molecules in mono- and bis-complexes are difficult to be obtained due to the Cu(OH)_2_ precipitation above pH 7.

## 3. CONCLUSIONS

Since Cu(II) coordination may become an important factor of sinefungin-induced cellular toxicity, the stability constants for the Cu(II)-sinefungin system were evaluated. They were compared to the analogous values obtained for the Cu(II)-ornithine species and appeared to be slightly lower. Despite the decrease in stability of cupric complexes of sinefungin, this ligand offers higher diversity of potential binding sites for copper(II) coordination process. Since the potentiometric titrations did not yield any data above pH 7 due to precipitation, it was not possible to describe the studied system at the physiological pH. However, we evaluated that, at pH 7, copper ions are completely bound by sinefungin molecules. In these conditions, two kinds of complexes coexist in water solution. They share the same coordination pattern of sinefungin (ornithine donor atoms), but the difference is in the amount of ligands engaged in complexation. Furthermore, DFT studies suggest that N_1_ nitrogen atom from adenine moiety also participates in the coordination. The calculations results propose that this process occurs through water molecule (*outer-sphere*). The *syn*-*anti* conformation equilibrium in complexed and free sinefungin depends on the protonation state and on the number of intramolecular hydrogen bonds.

## 4. EXPERIMENTAL SECTION

### 4.1. Materials

Sinefungin and HNO_3_ were purchased from Sigma Chemical Co.(MO, USA). NaClO_4_, Cu(ClO_4_)_2_, KNO_3_, and NaOH were obtained from Merck KGaA, (Darmstadt, Germany). Ethanediol was purchased from POCH S.A. (Gliwice, Poland).

### 4.2. Potentiometric titrations

Potentiometric titrations of sinefungin and its complexes with Cu(II) ions in the presence of 0.1 M KNO_3_ were performed at 25°C, within the pH range 2.8–10.5 (Molspin automatic titrator, Molspin Ltd, Newcastle upon Tyne, UK) with CO_2_-free 0.09997 M NaOH as titrant. Changes in pH were monitored with a combined glass-Ag/AgCl electrode (Russell pH Ltd., ThermoRussell CMAW 711, Fife, UK), calibrated daily in hydrogen ion concentrations by HNO_3_ titrations [57]. Sample volumes of 2 Ml were used. Ligand concentration was 0.7 MM, and a metal to ligand molar ratio of 1 : 2 was used. These data were analyzed using SUPERQUAD program [58]. Standard deviations computed by Superquad refer to random errors only.

### 4.3. Electronic absorption spectroscopy

The electronic absorption spectra were recorded at 25°C on a Cary 50 Bio spectrophotometer (Varian Inc., CA, USA) over the spectral range 190–900 nm, in 1 or 0.1 cm cells. The metal to ligand molar ratio was 1 : 2, and the concentration of the former one was 1.5 MM. The measurements were done in the presence of 0.1 M NaClO_4_, due to its transparency in far UV.

### 4.4. Circular dichroism spectroscopy

The CD spectra were recorded at the same conditions as UV-Vis measurements, on the Jasco J-715 spectropolarimeter (JASCO, Japan Spectroscopic Co., Hiroshima, Japan), over the ranges 190–350 nm, using 0.1 cm cells, and 300–800 nm, using 5 cm cells. The metal-ligand molar ratio and the concentration were the same as in UV-Vis measurements. Spectra were expressed in terms of $\Delta \varepsilon =\varepsilon_{\text{l}}-\varepsilon_{\text{r}}$, where $\varepsilon_{\text{l}}$ and $\varepsilon_{\text{r}}$ are molar absorption coefficients for left and right circularly polarized light, respectively.

### 4.5. Electron paramagnetic resonance

The spectra of the Cu(II) complex with sinefungin were recorded at 77 K on a Bruker ESP 300E spectrometer (Karlsruhe, Germany) at the X-band frequency (9.3 GHz). Ethanediol-water (1 : 2) was used as a solvent to obtain homogeneity of frozen samples. Samples concentrations were the same as those applied in other spectroscopic measurements.

### 4.6. Molecular modeling

All calculations were performed without any symmetry constraints with the use of Gaussian 03 program [59]. The density functional theory (DFT) method was used with the B3LYP hybrid functional [60–62]. In the calculations, the PCM solvent model was used [63], with water as the solvent. In the first step, geometries optimization was performed in the basis 6–31 G* for C, N, O, H atoms, and 6–311 G* for copper ion. The vibrational frequencies were calculated every time to confirm that the obtained structures correspond to local minima on the potential energy surface. Furthermore, the thermal corrections, for all optimized considered structures, were calculated. The obtained structures were used as inputs, and the energies of the optimized geometries were recalculated with the PCM model in a larger basis sets: 6–311 + G* for N, C, O, H in the case of free sinefungin molecule, 6–311 + G* for Cu, 6–31 + G* for N, C, O, and 6–31 + G** for H in the case of copper complexes. The data in tables contain values calculated with larger basis. The molecular structures were depicted by the program MOLDEN [64], and electrostatic potentials were drawn by the program gOpenMol [65].

Conformations of sinefungin in various protonation states were investigated, namely, H_4_L^3+^, H_3_L^2+^, H_2_L^+^, ${\text{HL}}^{\pm }$, and L^−^, where L^−^ stands for the fully deprotonated SFG molecule. In each case, optimization was done for the *syn* and *anti* conformations of the adenosine moiety. In the cases of H_3_L^2+^, H_2_L^+^, and ${\text{HL}}^{\pm }$, water molecules were added in the vicinity of −COO^−^ and neighboring −${\text{N}{\text{H}}_{3}}^{+}$ groups to prevent proton jumps between them, whereas the calculations for H_4_L^3+^ and L^−^ were performed without additional water molecules since the mentioned proton jump is not observed.

Geometry optimization of CuH_2_L^3+^ was performed for the coordination modes consistent with the experimental data, involving copper(II) coordination to the carboxylate oxygen and adenine nitrogen. Twelve water molecules were used in the optimization. In the considered structures, Cu(II) ion is coordinated by five or four water molecules. Water molecules, not entering the coordination sphere, are located around this sphere or H-bonded to polar groups in the SFG molecule. Also, the calculations for CuHL^2+^ complex species were performed with twelve water molecules. Four water molecules are directly bound to Cu(II), the remaining ones are located around the first coordination sphere. *Syn* and *anti* conformations of adenine were considered. For the ${\text{Cu}{\text{H}}_{2}{\text{L}}_{2}}^{2+}$, eight water molecules were included around the first coordination sphere of copper ion. Other six-coordinated complexes were also considered, in which, copper is additionally bound by N_7_ or N_1_ of adenine, either directly (*inner-sphere*) or indirectly (*outer-sphere*) through a water molecule.

## Figures and Tables

**Table 1:** The protonation constants of sinefungin (L) and the stability constants of its Cu(II) complexes with the process sites indicated. The analogous values for ornithine are included for comparison.

Species	log⁡β	${\text{pK}}_{\text{a}}$	Deprotonation site
H_4_L^3+^	24.90(1)	3.15	–COOH
H_3_L^2+^	21.75(1)	3.83	–N_1_(H^+^)
H_2_L^+^	17.918(9)	8.355	–${{\text{N}}_{\alpha }{\text{H}}_{3}}^{+}$
${\text{HL}}^{\pm }$	9.563(8)	9.563	–${{\text{N}}_{\delta }{\text{H}}_{3}}^{+}$

H_3_(Orn)^2+^	21.21^a^	1.98	–COOH
	21.02^b^	1.75	
H_2_(Orn)^+^	19.23^a^	8.74	–${{\text{N}}_{\alpha }{\text{H}}_{3}}^{+}$
	19.27^b^	8.75	
H(Orn)^±^	10.49^a^	10.49	–${{\text{N}}_{\delta }{\text{H}}_{3}}^{+}$
	10.52^b^	10.52	

CuHL^2+^	15.83(2)	—	—
${\text{Cu}{\text{H}}_{2}{\text{L}}_{2}}^{2+}$	31.12(2)	—	—

CuH(Orn)^2+^	17.8^a^	—	—
	17.812^b^	—	—
${\text{Cu}{\text{H}}_{2}{\left(\text{Orn}\right)}_{2}}^{2+}$	34.48^a^	8.98	—
	34.448^b^	—	—
${\text{Cu}\text{H}{\left(\text{Orn}\right)}_{2}}^{+}$	25.50^a^	9.97	—
Cu(Orn)_2_	15.53^a^	—	—

^a^[47], ^b^[48].

**Table 2:** Relative Gibbs free energies, Boltzmann factors (in percent), energy of solvation, and dihedral angle defining the positions of adenine for the SFG molecules.

	ΔG [kcal/mol]	${\text{E}}_{\text{solv}}$ [kcal/mol]	%^a^	∠O–C–N–C
H_4_L^3+^				

I (*anti*)	0.8	–322.5	20	183
II (*syn*)	0.0	–325.7	80	76

H_3_L^2+^				

III (*anti*)	0.8	–168.1	16	188
IV (*syn*)	0.0	–170.7	56	71
V	0.4	–170.8	28	55

H_2_L^+^				

VI (*anti*)	4.0	−83.0	0	170
VII (*syn*)	0.0	−89.7	100	68

HL				

VIII (*anti*)	0.0	−47.0	84	169
IX (*syn*)	1.0	−52.7	16	75

L^−^				

X (*anti*)	0.0	−79.7	96	174
XI (*syn*)	1.9	−87.4	4	76

^a^Values calculated from the Gibbs free energies.

**Table 3:** Relative Gibbs free energies, Boltzmann factors (in percent), and selected geometrical parameters for Cu(II)-SFG complexes.

	ΔG [kcal/mol]	%	r [Å] Cu–${\text{O}}_{\left(\text{COO}\right)}$	r [Å]Cu–N	r [Å] Cu–${\text{O}}_{\left({\text{H}}_{2}\text{O}\right)}$	∠O–C–N–C	∠Cu–N–C–C
CuH_2_L^3+^							

XII	—	100	—	2.02 (N_1_)	2.05	55	—
					1.97		
					1.96		
					2.48		
					2.49		

CuHL^2+^							

XIII (*anti*)	—	100	1.92	2.02 (N_*α*_H_2_)	2.04	169	—
					1.95		
					2.36		
					2.47		

${\text{Cu}{\text{H}}_{2}{\text{L}}_{2}}^{2+}$							

XIV (*syn*)	0.0	88	1.97	2.02	2.42	51	153
			1.97	2.01	2.51	52	−152
XV (*syn*)	3.9	0	2.01	2.02	2.46	63	−155
			1.99	2.00	2.41	58	−161
XVI (*syn*)	1.2	12	1.95	2.02	2.52	63	−173
			1.95	2.03	2.44	61	151
